# Sharpening and tightening surgical scissors

**Published:** 2023-12-01

**Authors:** Ismael Cordero

**Affiliations:** 1 Clinical Engineer, Philadelphia, USA.

*First published in Issue 76, 2011*

Surgical scissors consist of a pair of metal blades, pivoted so that the sharpened edges of each blade slide against each other when the handles opposite to the joint or pivot are closed.

The cutting edge of each blade is where the inner surface and the cutting surface meet (Figure 1). The two cutting edges cut as they slide over each other. The angle of the cutting surface is usually between 0 and 15 degrees from the horizontal. Scissors with a very steep angle (nearer 15 degrees) are extremely sharp and are meant for cutting soft tissues such as conjunctiva. Scissors with a less steep angle are meant for cutting harder tissues.

**Figure 1:**
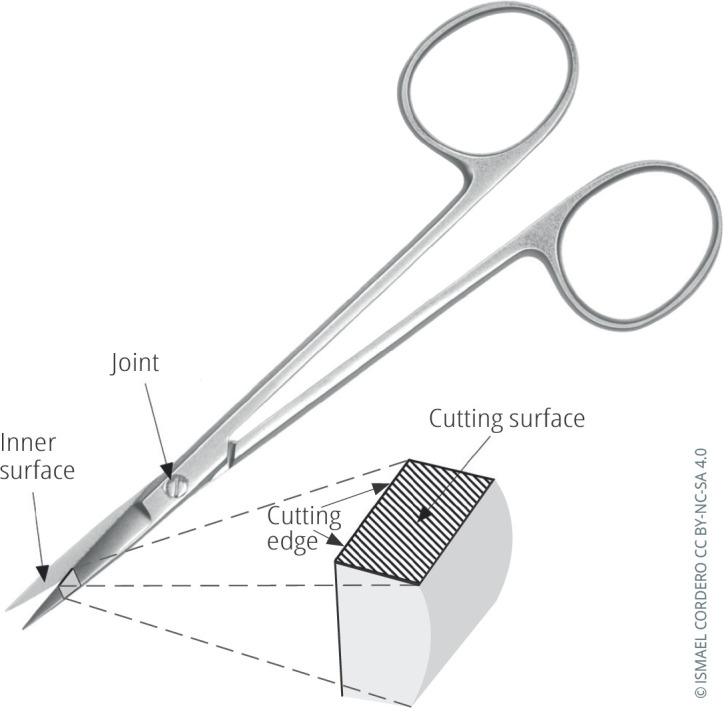
The cutting edge of each blade is where the inner surface and the cutting surface meet.

With repeated use, the sharp cutting edges become rounded and pits or gaps can appear, making the scissors blunt. These pits will be visible as changes in the reflection when you examine the cutting surfaces in bright light.

The sharper the cutting edge, the quicker the scissors will become blunt. You should never use scissors to cut material that the scissors are not suitable for, or they will become blunt very quickly. If blunt scissors are used, the tissue will be clasped instead of cut, resulting in contusion of the tissue and ineffectual wound healing.

## Testing the scissors

1. Stretch a piece of cotton wool so that a small, straight piece is formed, with the width equal to the length of the scissor blades.
2. Cut this piece using the whole length of the scissors.
3. Gently pull the cotton wool out while the scissors are still in the closed position. If the scissors are working well, there should be a nice, straight cut in the cotton wool. If not, and the scissors clasp the cotton wool, this may be because the scissor blades are blunt or because the joint is too loose.

## Sharpening the scissors

A pair of scissors is sharpened by filing off a very thin layer of the cutting surface to create a new cutting edge.

You may use a small, fine triangular file; however, if you have access to a triangular sharpening stone (800-1,200 grit) you will achieve even better results.

To obtain the smoothest surface possible, place a few drops of sewing machine oil on the sharpening stone.

**Note:** Always sharpen scissors by filing along the cutting surface, never on the inner surface.

**Figure 2:**
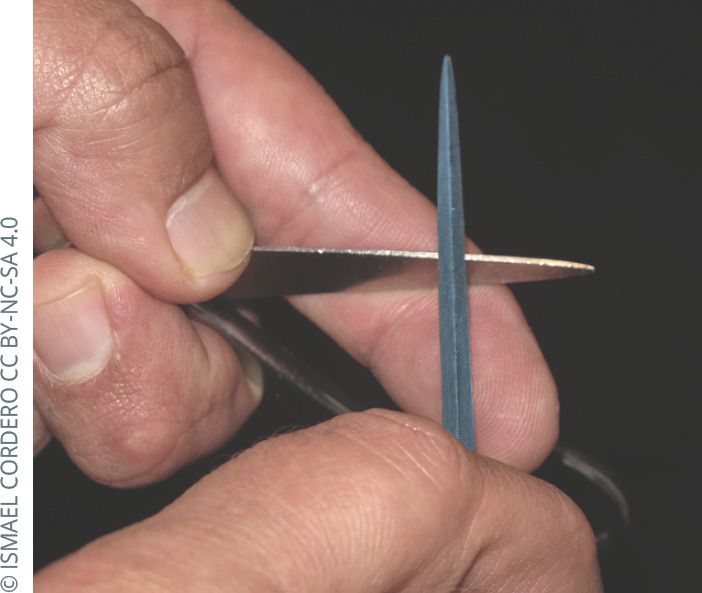
Sharpening scissors.

1. Hold the scissors firmly in one hand (your left hand if you are right-handed, and vice versa), with the back of one blade resting on the end of your index finger and the cutting surface visible (Figure 2). Keep the joint open by pressing your thumb against the hand-piece of the scissors.
2. Place a bright desk lamp at the same height as your eyes. Let the
3. light reflect on the cutting surface. Rotate the scissors slowly in both directions. When the reflection is at its brightest, the surface is horizontal. If you keep the sharpening stone horizontal as well, you will preserve the original angle.
4. Always start sharpening at the tip of the instrument, to prevent rounding off the tip. Make a gentle stroke in a forwards direction (away from you) and simultaneously towards the joint. Make sure to cover the whole surface with each stroke so that you do not create different levels along the length of the blade. Do not apply too much force. The repetition of the movement is what sharpens the scissors.
5. Repeat until most of the pit reflections are gone. If the pits are too deep, the amount that has to be filed off to get rid of them may be too large and you run the risk that the cutting surfaces of the scissor blades no longer touch each other. It may be necessary to remove such scissors from circulation.
6. After sharpening, a burr (an accumulation of filed metal) may be formed on the inner surface. This burr has to be removed. If not, it will damage the cutting edge on the opposite side during cutting. You can remove any burs by scratching them off with your fingernail.
7. Repeat the procedure for the other scissor blade. Always sharpen both blades.
8. Clean the scissors thoroughly after sharpening. Any remnants of oil and metal on the instrument can cause inflammation in the eye.

## Tightening a loose joint

Another reason why scissors may not cut properly is a loose joint. If the screw or rivet is not tight, the distance between the two inner surfaces will be too large, causing the cutting surfaces to not touch each other. As a result, tissues will be clasped instead of cut.

1. Place the scissors on a flat, hard surface.
2. Close the scissors so that the blades are top of each other.
3. If the joint has a screw, then tighten it. If it has a rivet, then proceed to the next step.
4. Place the tip of a pin punch on top of the rivet head, keeping the pin punch perpendicular to the scissors.
5. While holding the scissors down, have someone else hit the top of the pin punch with a small hammer.
6. Test the scissors after every hit, to prevent them from becoming too tight.

